# Effect of vitamin D status on normal fertilization rate following in vitro fertilization

**DOI:** 10.1186/s12958-019-0500-0

**Published:** 2019-07-18

**Authors:** Xuemei Liu, Wei Zhang, Yanping Xu, Yongli Chu, Xinrong Wang, Qian Li, Zhi Ma, Zhenteng Liu, Yanling Wan

**Affiliations:** 1 Reproductive Medicine Center, Yantai Yuhuangding Hospital, Affiliated Hospital of Qingdao University, Yantai, Shandong China; 2 Department of Obstetrics and Gynecology, Yantai Yuhuangding Hospital, Affiliated Hospital of Qingdao University, Yantai, Shandong China; 3 Scientific Research Office, Yantai Yuhuangding Hospital, Affiliated Hospital of Qingdao University, Yantai, Shandong China

**Keywords:** Vitamin D, Clinical pregnancy rate, Live birth rate, In vitro fertilization

## Abstract

**Background:**

Vitamin D plays critical role in the female reproductive system. It seems that vitamin D is associated with clinical pregnancy outcomes of assisted reproductive technologies (ART), but its role remains elusive. This study is aimed to establish whether vitamin D is associated with clinical outcomes of in vitro fertilization (IVF).

**Methods:**

The cross-sectional study was carried out from January 1st 2017 to December 31st 2017. A total of 848 patients who had indications for IVF were enrolled. The patients were classified by serum 25 (OH) D quartiles. The outcome parameters of IVF were compared in each group, including normal fertilization rate, high quality embryo rate, clinical pregnancy rate, implantation rate and live birth rate.

**Results:**

The median 25 (OH) D concentration was 15.25 ng/ml. Serum 25 (OH) D levels in women varied with the seasons. We found that serum 25 (OH) D levels were higher in autumn than other seasons, and the lowest level occurred in spring. Follicular fluid (FF) vitamin D levels were positively correlated with serum vitamin D levels (*r* = 0.85, *P* < 0.001). The levels of FF vitamin D were significantly higher than the levels of serum vitamin D (*P* < 0.001). Normal fertilization rates were significantly different among four groups (*P* = 0.007). The group of women with the highest serum 25 (OH) D levels had the highest normal fertilization rate. However, the clinical pregnancy rate, implantation rate and live birth rates were not significantly different among the four groups when the age, BMI, AMH, seasons of blood drawing, COH protocol, high quality embryo rate and number of embryos transferred were adjusted. In addition, we found that serum 25 (OH) D levels were significantly higher in patients received IVF than patients received R-ICSI (*P* = 0.013).

**Conclusions:**

Among Chinese women, lower serum vitamin D levels are associated with a lower fertilization rate in IVF. However, vitamin D level was not associated with the clinical pregnancy and live birth rate following IVF.

**Electronic supplementary material:**

The online version of this article (10.1186/s12958-019-0500-0) contains supplementary material, which is available to authorized users.

## Background

Vitamin D is a fat-soluble steroid hormone. It has an important role in Calcium-Phosphate (Ca-P) homeostasis and bone metabolism [1, 2]. Vitamin D comes mainly from sunlight. Only a small amount comes from diet. Vitamin D deficiency has been emerging in recent two decades among all racial groups [3]. Vitamin D insufficiency is endemic because of poor sunlight exposure, lifestyle, use of sunscreens, low dietary intake, and/or increased body mass index (BMI) [4, 5]. Vitamin D deficiency has been considered a public health problem in all over the world. Low vitamin D concentrations have been associated with an increased risk of many chronic diseases, including autoimmune disease, diabetes, cardiovascular disease, obesity and cancer [6]. Recently, poor vitamin D status verified in 20–52% of women of reproductive years, suggests that vitamin D has an important role in female reproduction [7–9].

Vitamin D exerts effect by binding with the nuclear vitamin D receptor. Vitamin D receptors have been identified in many tissues, including the ovary, uterus, placenta and the pituitary [10]. These data indicate that vitamin D may be an important regulator of the reproductive system. The importance of vitamin D in reproduction is evident from animal studies. Vitamin D deficiency could cause uterine hypoplasia, reduced fertility, hypogonadism and impaired folliculogenesis [1, 11–13]. In humans, vitamin D has an important role in placental function [14]. Vitamin D deficiency is associated with poor placentation, fetal growth restriction, gestational hypertension and pre-eclampsia [14]. In addition, vitamin D plays critical role in oocyte development, ovarian steroidogenesis, production of anti-Mullerian hormone (AMH), endometrial receptivity, and others [15, 16]. It was supposed that vitamin D is associated with clinical outcomes of assisted reproductive technologies (ART).

So far, there were many studies investigating the association between vitamin D level and the clinical outcomes of ART, but the data are inconclusive. Some studies found that vitamin D could improve the outcomes of ART [17–24]. In contrast, other studies reported that vitamin D has no effect on the outcomes of ART [25–30]. Hence, the role of vitamin D in ART is still unclear. To solve this problem, we set up a large retrospective cross-sectional study to evaluate the association between serum levels of 25(OH) D and outcomes of ART in Chinese women.

## Methods

### Trial design and participants’ characteristics

This was a retrospective cohort study. We have retrospectively reviewed the data of all patients, who underwent their in vitro fertilization (IVF) cycle at Yantai Yuhuangding Hospital from January 1st 2017 to December 31st 2017. The study protocol was approved by the Ethical Committee of Yantai Yuhuangding Hospital. The study conformed to the “Declaration of Helsinki for Medical Research involving Human Subjects”. Patient characteristics and cycle parameters were obtained from patient medical records.

Only one IVF cycle of each patient was selected within the study time frame. None of the patients received vitamin D supplements. Patients were excluded if they were diagnosed with premature ovarian in sufficiency or if they were treated with intracytoplasmic sperm injection (ICSI) or rescue ICSI (R-ICSI). To avoid confounders, women whose plasma total 25 (OH) D measurements exceeded 4 weeks prior to entering their IVF cycle were excluded from the study, since the season impacts on the serum 25 (OH) D concentrations in Yantai.

### Procedure of IVF

All patients undergoing IVF cycles receive luteal phase gonadotrophin-releasing hormone (GnRH) agonist protocol or GnRH-antagonist protocol. In luteal-phase gonadotrophin-releasing hormone (GnRH) agonist protocol, low- or regular- euprolide was used to make pituitary desensitization beginning in the luteal phase. In GnRH-antagonist protocol, GnRH-antagonist ganirelix was used when the dominant follicle reached 12 mm in diameter, estradiol concentrations were>150 pg/ml and/or luteinizing hormone concentrations were >10 IU/L. During gonadotrophin stimulation, serum estradiol, progesterone and luteinizing hormone, follicle size measurements, and endometrial thickness were monitored until the day of human chorionic gonadotropin (hCG) trigger injection. When two leading follicles reached a mean diameter of 18 mm, hCG was given to trigger ovulation. Oocytes were retrieved transvaginally 34–36 h after hCG administration. IVF was performed. Ultrasound-guided fresh embryo transfer was performed on the third day after oocyte retrieval, and excess viable embryos were cultured to blastocyst stage and cryopreserved for subsequent frozen embryo transfer (FET) cycles. The number of embryos transferred was one or two depending on the number of available embryos. Fresh embryo transfer was cancelled if the embryo and endometrium were not synchronous or women had a high risk of ovarian hyperstimulation syndrome and factors seriously affecting embryo implantation. The obtained embryos were graded depending on published criteria [31]. The embryos of grade 1 or 2 were considered as high-quality embryos. The luteal phase was supported with 200 mg progesterone (Utrogest™ 200, Besins-Iscovesco, France) vaginal medication three times daily from the day of oocyte retrieval. A quantitative pregnancy test (serum β-hCG based) was performed on the 14th day after embryo transfer. In terms of pregnancy, a transvaginal ultrasound was performed after 4 weeks from the embryo transfer. Clinical pregnancy was confirmed if the fetal heartbeat was observed by transvaginal ultrasound. Live birth was defined as the birth of a neonate.

### Serum 25 (OH) D measurement

Total 25 (OH) D was measured before entering IVF cycle. The measurement of total 25 (OH) D levels was performed using a chemiluminescence immunoassay (DiaSorin). The intra- and inter- assay coefficients of variations were 10 and 15%, respectively.

### Serum and follicular fluid (FF) collection and 25 (OH) D measurement

Sixty-two serum samples were obtained on the oocyte retrieval day and were kept frozen at − 80 °C. FF had been also collected from the same 62 patients. FF aspiration was performed from the first follicle on oocyte retrieval day. FF was centrifuged at 3000 g for 10 min. The supernatant was stored at − 80 °C for subsequent assay. Serum and FF 25 (OH) D levels were measured by a chemiluminescence immunoassay (DiaSorin).

The levels of serum vitamin D varied significantly in different seasons because of the different exposure to sunlight (Additional file 1: Table S1) [32]. Therefore, the season of blood drawing was included in the study. Each season lasted 3 months: Spring: February 1–April 30; Summer: May 1–July 31; Autumn: August 1–October 31; Winter: November 1–January 31.

### Study design

All patients were assigned to quartiles depending on the levels of vitamin D before entering the IVF cycle. The demographic and reproductive characteristics were calculated by descriptive statistics according to quartiles of vitamin D concentrations. We evaluated the association of serum 25 (OH) D concentrations in quartiles and treatment outcomes of IVF cycle while adjusting for potential confounding factors.

### Main outcome measures

The primary outcome was clinical pregnancy rate. It was defined as the presence of an intrauterine sac confirmed by ultrasound at 6 weeks of gestation. Secondary outcomes were: live birth rate, implantation rate and (2 PN) fertilization rate.

### Statistical methods

The continuous variables were presented as mean ± SD, and the proportions were presented as percentages. The Kruskal-Wallis test was used for continuous variables. The chi-square test was used for categorical variables.

Receiver operating characteristic (ROC) curves were used to analyze the predictive value of serum vitamin D levels on clinical pregnancy rate, live birth rate and fertilization rate. Low fertilization rate (LFR) was defined as normal fertilization rate below 30% as it was a clinical standard for ICSI to promote normal fertilization rate. Spearman’s test was used to evaluate the correlation between serum vitamin D levels and FF vitamin D levels.

A univariate and multivariate logistic regression analysis was applied to analyze the relationship of vitamin D and clinical outcomes of IVF. The variables that were evaluated as potential confounders included: age, BMI, AMH, type of infertility, previous pregnancy, seasons when samples were taken, the type of treatment protocol, duration of stimulation, E_2_ levels on the day of hCG, number of oocytes retrieved, number of mature oocytes, the percentage of top quality embryos, endometrial thickness, serum progesterone levels on the day of hCG and the number of embryo transferred.

The data were analyzed by use of the SPSS-12.0 software. *P*<0.05 was considered to be statistically significant.

## Results

**Fig. 1:**
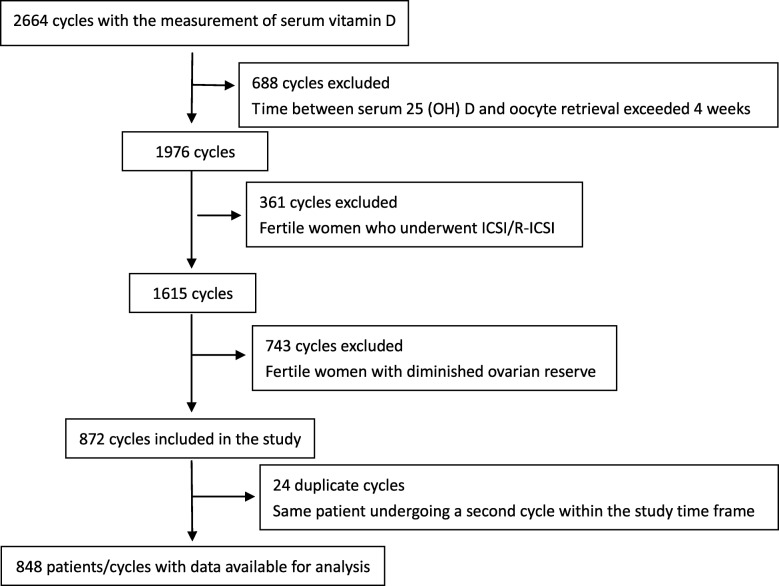
Patient selection process

Overall, 848 patients were included in this study (Fig. 1). Of the 848 fresh cycles with oocyte retrieval, 487 cycles had fresh embryo transfer. The median (minimum, maximum) 25 (OH) D concentration was 15.25 ng/ml (4.92–39.51 ng/ml).

**Fig. 2:**
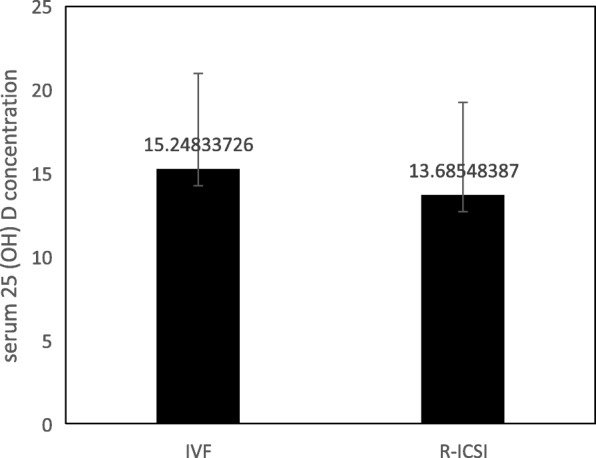
Serum 25 (OH) D concentration were significantly higher in IVF group than R-ICSI group (*P* = 0.013)

Table 1 depicts patients and IVF cycle characteristics according to serum 25 (OH) D quartiles. The levels of vitamin D were inversely related to BMI and AMH, though this difference was not statistically significant (*p* = 0.099 and *p* = 0.134, respectively). 25 (OH) D concentrations were significantly related to seasons when the samples were taken (*P*<0.001). Serum 25 (OH) D levels were higher in autumn than other seasons, and the lowest level occured in spring (Additional file 1: Table S1). Vitamin D status was not associated with age, infertility diagnosis, duration of infertility, previous pregnancy, stimulation protocol, ovarian stimulation parameters (duration of stimulation, peak estradiol levels, progesterone on HCG day, endometrial thickness or number of mature oocytes, as noted in Table 1), high quality embryo rate, blastocyst formation rate or number of embryos transferred (p>0.05). 25 (OH) D concentrations were inversely related to the number of larger follicles (≥16 mm on HCG day) and number of oocytes, though this difference was not statistically significant (*p* = 0.083 and *p* = 0.078, respectively). However, the levels of vitamin D were positively related to normal fertilization rates (*p* = 0.007). A similar relationship was observed between vitamin D status and the fertilization rates (*P*<0.001) after adjusting for potential confounders (age, BMI, AMH, type of infertility, previous pregnancy, seasons when sample were taken, the type of treatment protocol, duration of stimulation, E_2_ levels on the day of hCG, number of larger follicles, number of oocytes retrieved and number of mature oocytes).

**Table 1:** Patients and IVF cycle characteristics by serum 25 (OH) D quartiles

Parameter	Group1	Group2	Group 3	Group 4	*P* value
Number of cycles	216	210	211	211	
Serum 25(OH) D (ng/ml)					
Median	9.04	13.67	16.20	23.22	
Range	4.92–11.20	11.25–14.00	14.10–18.60	18.70–39.51	
Age(y)	31.65 ± 3.32	31.86 ± 3.94	31.31 ± 3.44	31.88 ± 3.69	0.331
Duration of infertility(y)	3.53 ± 2.19	3.85 ± 2.76	3.68 ± 2.31	3.61 ± 2.38	0.543
BMI (kg/m^2^)	23.93 ± 3.61	23.37 ± 3.30	23.16 ± 3.28	23.46 ± 3.33	0.099
AMH (ng/ml)	7.21 ± 5.50	6.05 ± 4.37	6.93 ± 5.61	6.68 ± 5.51	0.134
bFSH (mIU/ml)	6.75 ± 1.77	7.04 ± 2.03	6.88 ± 1.95	6.57 ± 1.68	0.063
Season of blood drawing					
Spring (n, %)	55,25.5%	39,18.6%	25,11.8%	24,11.4%	<0.001*
Summer (n, %)	78,36.1%	71,33.8%	55,26.1%	41,19.4%	
Autumn (n, %)	22,10.2%	43,20.5%	76,36.0%	74,35.1%	
Winter (n, %)	61,28.2%	57,27.1%	55,26.1%	72,34.1%	
Previous pregnancy					
NO (n, %)	106 (49.1%)	87 (41.4%)	111 (52.6%)	103 (48.8%)	0.134
Yes (n, %)	110 (50.9%)	123 (58.6%)	100 (47.4%)	108 (51.2%)	
Infertility diagnosis					
Male factor (*n*, %)	5 (2.3%)	11 (5.2%)	10 (4.7%)	10 (4.7%)	0.837
Tubal factor (*n*, %)	135 (62.8%)	131 (62.4%)	122 (57.8%)	125 (59.2%)	
Mixed (*n*, %)	24 (11.2%)	28 (13.3%)	26 (12.3%)	28 (13.3%)	
Endometriosis (*n*, %)	7 (3.3%)	11 (5.2%)	13 (6.2%)	14 (6.6%)	
Anovulatory (*n*, %)	41 (19.1%)	27 (12.9%)	37 (17.5%)	32 (15.2%)	
Unexplained (*n*, %)	3 (1.4%)	2 (1.0%)	3 (1.4%)	2 (0.9%)	
COH protocol					
Luteal phase agonist (*n*, %)	151 (69.9%)	143 (68.1%)	159 (75.4%)	158 (74.9%)	0.250
Antagonist (*n*, %)	65 (30.1%)	67 (31.9%)	52 (24.6%)	53 (25.1%)	
Duration of stimulation (d)	9.06 ± 2.17	9.04 ± 2.34	8.81 ± 2.20	9.05 ± 2.50	0.631
Follicle≥16 mm on HCG day	10.26 ± 4.94	9.01 ± 4.73	9.86 ± 5.55	9.68 ± 5.02	0.083
Endometrial thickness on HCG day (mm)	11.62 ± 2.71	11.30 ± 2.21	11.36 ± 2.32	11.49 ± 2.45	0.536
Progestrone on HCG day (ng/ml)	0.86 ± 0.28	0.90 ± 0.29	0.94 ± 0.34	0.94 ± 0.32	0.143
Peak estradiol (pg/ml)	3737.49 ± 2178.84	3398.98 ± 2227.44	3593.33 ± 2352.79	3569.79 ± 2231.67	0.540
Oocytes retrieved (*n*)	11.47 ± 5.53	10.12 ± 4.78	10.79 ± 5.43	10.68 ± 5.56	0.078
Mature oocytes (*n*)	9.45 ± 4.80	8.40 ± 4.14	9.05 ± 4.66	8.97 ± 4.54	0.119
Normal fertilization rate (%)	69.62 ± 17.85	71.69 ± 19.92	73.82 ± 18.36	75.43 ± 17.39	0.007* <0.001^a*^
High quality embryo rate (%)	67.5%	67.2%	65.3%	67.7%	0.465
Blastocyst formation rate (%)	66.86 ± 28.60	62.48 ± 33.21	65.84 ± 31.27	67.96 ± 28.87	0.332
Embryos transferred (*n*)	1.93 ± 0.26	1.92 ± 0.28	1.97 ± 0.18	1.95 ± 0.22	0.340

*Values are significantly different between groups (*P* < 0.05)

^a^Adjusted for maternal age, BMI, AMH, infertility diagnosis, season of blood draw, COH protocol, previous pregnancy, duration of stimulation, E2 levels on the day of hCG, number of larger follicles, number of oocytes retrieved and number of mature oocytes

**Fig. 3:**
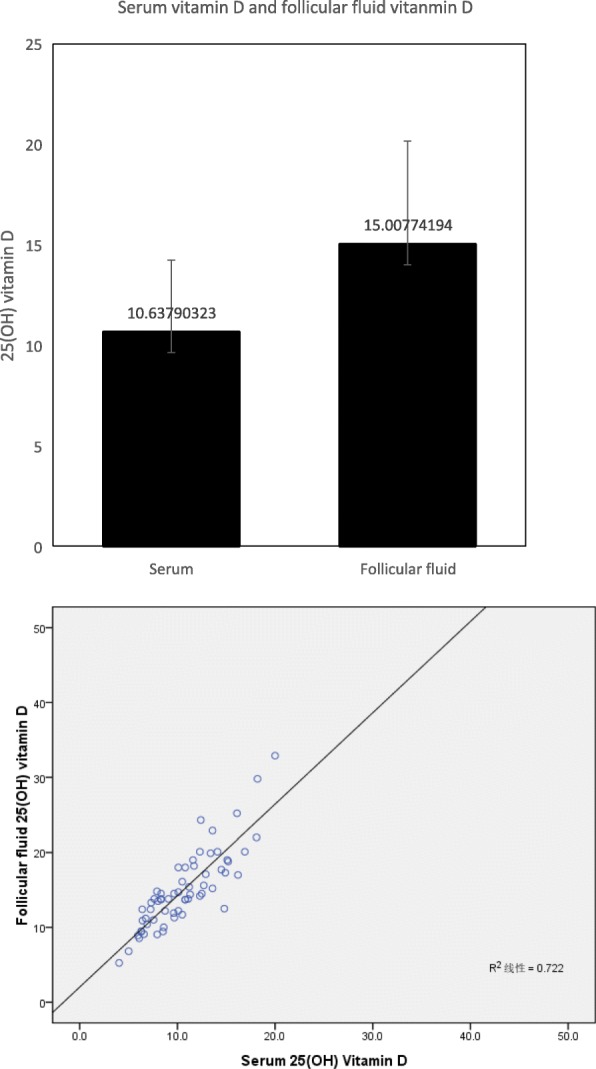
The relationship of follicular fluid vitamin D levels and serum vitamin D levels. Follicular fluid vitamin D levels were positively correlated with serum vitamin D levels (*r* = 0.85, *p* < 0.001). The levels of follicular fluid vitamin D were significantly higher than the levels of serum vitamin D (*P* < 0.001)

To further determine the effect of vitamin D on fertilization, we compared vitamin D levels in patients treated with IVF and R-ICSI. We found that serum vitamin D levels were significantly higher in patients treated with IVF than R-ICSI (*p* = 0.013) (Fig. 2). In addition, we observed the relationship of follicular fluid vitamin D levels and serum vitamin D levels. The results showed that FF vitamin D levels were positively correlated with serum vitamin D levels (*r* = 0.85, *p* < 0.001). The levels of FF vitamin D were significantly higher than the levels of serum vitamin D (*P* < 0.001) (Fig. 3).

Table 2 depicts the patient and IVF cycle characteristics by pregnancy outcome. Live birth was associated with age (*P*<0.001), stimulation protocol (*p* = 0.027), endometrial thickness (*P* = 0.002), number of oocytes retrieved (*p* = 0.02), number of mature oocytes (*p* = 0.024) and high quality embryo rate (*p* = 0.001). AMH, peak estradiol, number of larger follicles and embryos transferred were greater in the live birth group, but they did not achieve statistical significance. Only age (*P*<0.001) and endometrial thickness (*P* = 0.008) were significantly associated with clinical pregnancy. The number of larger follicles, oocytes and embryos transferred were higher in the clinical pregnancy group, but they did not achieve statistical significance.

**Table 2:** Patients and IVF cycle characteristics by pregnancy outcome

Parameter	Pregnant	Not pregnant	*P* value	Live birth	No live birth	*P* value
Number of cycles	293	194		261	226	
Age(y)	31.29 ± 3.53	32.63 ± 3.65	<0.001*	31.12 ± 3.41	32.64 ± 3.71	<0.001*
Duration of infertility(y)	3.54 ± 2.26	3.77 ± 2.43	0.284	3.45 ± 2.15	3.84 ± 2.52	0.065
BMI (kg/m^2^)	23.25 ± 3.34	23.74 ± 3.46	0.116	23.28 ± 3.34	23.62 ± 3.46	0.271
AMH (ng/ml)	5.31 ± 3.76	4.83 ± 3.55	0.160	5.41 ± 3.82	4.79 ± 3.50	0.065
bFSH (mIU/ml)	6.98 ± 1.74	7.10 ± 1.88	0.475	6.93 ± 1.74	7.14 ± 1.87	0.208
Season of blood drawing						
Spring (*n*, %)	41,54.7%	34,45.3%	0.209	37,14.2%	4,12.5%	0.706
Summer (*n*, %)	83, 58.5%	59,41.5%		76,29.1%	7,21.9%	
Autumn (*n*, %)	83,68.0%	39,32.0%		74,28.4%	9,28.1%	
Winter (*n*, %)	86; 58.1%	62,41.9%		74,28.4%	12,37.5%	
Previous pregnancy						
NO (*n*, %)	141, 48.1%	89, 45.9%	0.628	129,49.4%	106,46.9%	0.587
Yes (*n*, %)	152, 51.9%	105, 54.1%		132,50.6%	120,53.1%	
Infertility diagnosis						
Male factor (*n*, %)	11,3.8%	6,3.1%	0.426	10,3.8%	6,2.7%	0.688
Tubal factor (*n*, %)	193,65.9%	125,64.4%		168,64.4%	150,66.4%	
Mixed (*n*, %)	41,14.0%	21,10.8%		38,14.6%	25,11.1%	
Endometriosis (*n*, %)	18,6.1%	11, 5.7%		16,6.1%	12,5.3%	
Anovulatory (*n*, %)	28,9.6%	27,13.9%		26,10.0%	29,12.8%	
Unexplained (*n*, %)	2,0.7%	4,2.1%		3,1.1%	4,1.8%	
COH protocol						
Luteal phase agonist (*n*, %)	228,77.8%	138,71.1%	0.108	207,79.3%	159,70.4%	0.027*
Antagonist (*n*, %)	65,22.2%	56,28.9%		54,20.7%	67,29.6%	
Duration of stimulation (d)	9.08 ± 2.31	9.05 ± 2.06	0.908	9.06 ± 2.26	9.07 ± 2.16	0.962
Follicle≥16 mm on HCG day	7.74 ± 3.04	7.22 ± 2.96	0.059	7.78 ± 2.90	7.25 ± 3.13	0.055
Endometrial thickness on HCG day (mm)	11.75 ± 2.10	11.22 ± 2.24	0.008*	11.82 ± 2.09	11.21 ± 2.22	0.002*
Progestrone on HCG day (ng/ml)	0.92 ± 0.29	0.90 ± 0.33	0.578	0.92 ± 0.29	0.90 ± 0.33	0.332
Peak estradiol (pg/ml)	2602.72 ± 1142.74	2436.73 ± 1032.56	0.116	2625.10 ± 1114.09	2436.06 ± 1078.48	0.070
Oocytes retrieved (*n*)	8.39 ± 2.98	7.91 ± 3.28	0.094	8.50 ± 2.95	7.85 ± 3.25	0.020*
Mature oocytes (*n*)	7.03 ± 2.68	6.66 ± 2.99	0.156	7.15 ± 2.68	6.58 ± 2.92	0.024*
Normal fertilization rate (%)	72.55 ± 17.70	73.19 ± 20.05	0.710	72.22 ± 17.18	73.48 ± 20.23	0.456
High quality embryo rate (%)	59.5%	57.1%	0.199	58.4%	51.6%	0.001*
Blastocyst formation rate (%)	66.33 ± 31.39	62.75 ± 34.51	0.273	66.06 ± 31.59	63.61 ± 33.90	0.443
Embryos transferred (*n*)	1.96 ± 0.21	1.92 ± 0.28	0.082	1.96 ± 0.20	1.92 ± 0.27	0.081

*Values are significantly different between groups (*P* < 0.05)

As shown in Table 3, serum 25 (OH) D levels were not related to clinical pregnancy rates, implantation rates, abortion rates and live birth rates. Results though were similar after adjusting for potential confounding factors (age, BMI, AMH, type of infertility, previous pregnancy, seasons when samples were taken, the type of treatment protocol, duration of stimulation, number of oocytes retrieved, endometrial thickness, serum progesterone levels on the day of hCG and the number of embryo transferred).

**Table 3:** Serum 25 (OH) D concentrations in relation to clinical outcomes of IVF: univariate and multivariate logistic analysis

Outcomes	Group1	Group2	Group 3	Group 4
Number of cycles	216	210	211	211
Clinical pregnancy rate (%)	59.2%	57.9%	60.0%	63.5%
Univariate (OR(95%CI),P)	1	0.947 (0.567–1.581),0.836	1.035 (0.618–1.734),0.895	1.200 (0.718–2.006),0.486
Multivariate^a^ (OR(95%CI),P)	1	1.007 (0.578–1.752),0.981	0.988 (0.562–1.737),0.968	1.173 (0.674–2.043),0.573
Implantation rate (%)	42.9%	41.4%	44.5%	43.9%
Univariate (OR(95%CI),P)	1	0.941 (0.651–1.361),0.747	1.069 (0.741–1.541),0.722	1.043 (0.726–1.499),0.818
Multivariate^a^ (OR(95%CI),P)	1	0.958 (0.647–1.419),0.931	1.024 (0.690–1.518),0.907	1.021 (0.693–1.506),0.915
Abortion rate (%)	8.5%	10.0%	2.8%	8.8%
Univariate (OR(95%CI),P)	1	1.398 (0.459–4.260),0.556	0.637 (0.172–2.362),0.500	1.373 (0.463–4.070),0.567
Multivariate^a^ (OR(95%CI),P)	1	1.319 (0.365–4.768),0.673	0.732 (0.171–3.126)0.673	1.302 (0.373–4.544),0.679
Live birth rate (%)	53.3%	48.8%	56.7%	55.6%
Univariate (OR(95%CI),P)	1	0.833 (0.502–1.381),0.478	1.144 (0.688–1.903),0.604	1.094 (0.662–1.807),0.726
Multivariate^a^ (OR(95%CI),P)	1	0.876 (0.507–1.515),0.636	1.089 (0.624–1.901),0.765	1.078 (0.626–1.856),0.787

Values are significantly different between groups (*P* < 0.05)

^a^Adjusted for maternal age, BMI, AMH, type of infertility, previous pregnancy, seasons when samples were taken, COH protocol, duration of stimulation, number of oocytes retrieved, endometrial thickness, serum progesterone levels on the day of hCG and the number of embryo transferred

**Fig. 4:**
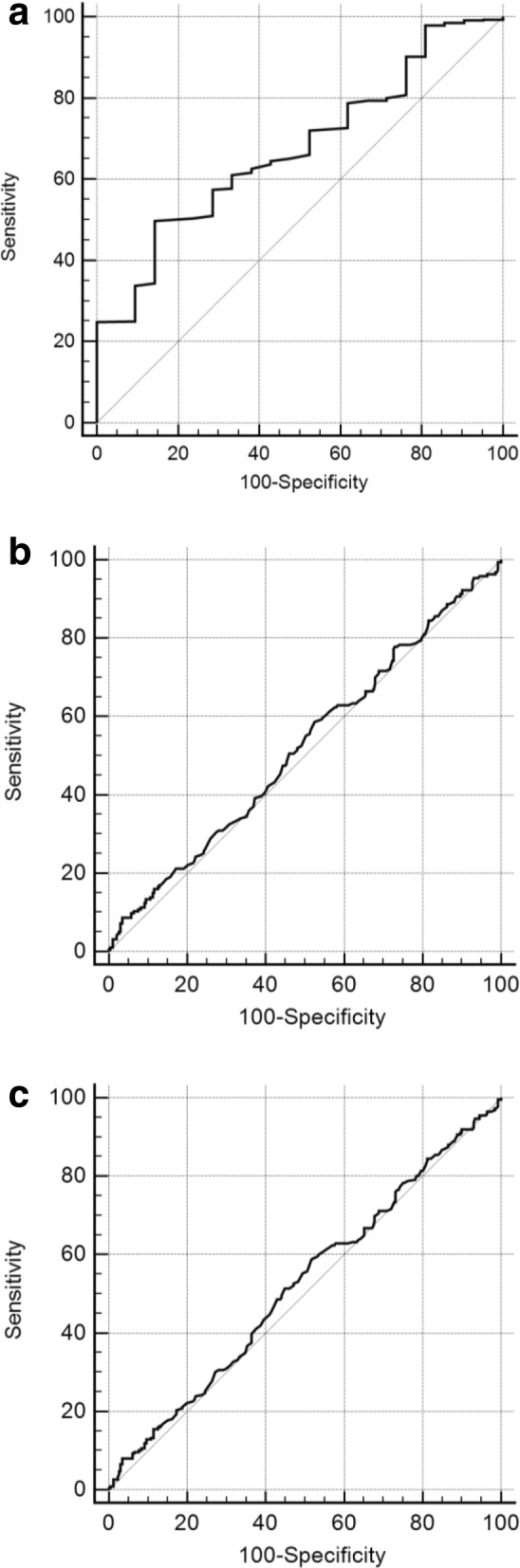
ROC curve and performance characteristics for total serum vitamin D measured in patients. **a** (normal fertilization rate): The area under the ROC curve for vitamin D was 0.674, *p* = 0.0007. **b** (clinical repgnancy rate): The area under the ROC curve for vitamin D was 0.521, *p* = 0.4251. **c** (live birth rate): The area under the ROC curve for vitamin D was 0.523, *p* = 0.3822

The predictive value of vitamin D regarding normal fertilization rate, pregnancy rate and live birth rate were analyzed by ROC curve (Fig. 4). After analyzing the ROC curve, optimal sensitivity and specificity at the cutoff point (14.1 ng/ml) were found to be 49.8 and 85.7% for the fertilization rate. The area under the ROC curve for vitamin D was 0.674 (*p* = 0.0007). However, for pregnancy rate and birth rate, the area under the ROC curve was 0.521 and 0.523 respectively, demonstrating poor performance for the prediction of pregnancy and live birth.

## Discussion

In this large retrospective cross-sectional study, 848 women underwent 848 IVF cycles. The serum 25 (OH) D concentrations were between 4.92 and 39.51 ng/ml. The median 25 (OH) D concentration was 15.25 ng/ml. Serum vitamin D levels significantly vary in different seasons because of the different exposure to sunlight. Serum 25 (OH) D levels were higher in autumn than other seasons, and the lowest level occurred in spring (Additional file 1: Table S1).

This study investigated the association between vitamin D and outcomes of IVF. Our results indicate that vitamin D was positively related to normal fertilization rate. Women in the highest quartile of vitamin D had a 5.81% higher normal fertilization rate than women in the lowest quartile. Moreover, serum vitamin D levels were significantly lower in patients treated with R-ICSI than those with IVF. FF vitamin D levels were positively correlated with serum vitamin D levels and were significantly higher than the levels of serum vitamin D. Therefore, we believe that vitamin D can affect fertilization. However, the role of vitamin D in fertilization remains unclear. As we all know, the process of fertilization includes two key steps: the interaction and penetration of the sperm to the zona pellucida, the fusion of the sperm and the oocyte membranes and triggering oocyte activation. In this study, we found these oocytes could be actived and could form embryos after R-ICSI. Therefore, vitamin D could affect fertilization by altering the capacity of interaction and penetration of the zona pellucid by the sperm. Some researchers found that vitamin D had effect on the development of follicle [24]. It may have been speculated that more 25 (OH) D are responsive to improve oocytes to reach a more mature stage prior to oocyte retrieval, resulting in a greater capability to achieve fertilization by improving the sperm interaction and penetration of the zona pellucida.

Our result of a positive relationship of vitamin D and normal fertilization rates is in accordance with previous works [33, 34]. In contrast, two studies showed an inverse association between 25 (OH) D and fertilization rates among women undergoing infertility treatment [26, 35]. However, these data of Aleyasin et al., are difficult to explain in the Iranian study because all participants were vitamin D deficient, except for one [26]. Though serum and FF vitamin D are strongly related to each other (Fig. 3, [26]), Ciepiela et al., evaluated vitamin D levels only from the dominant follicle [35]. Moreover, the sample size of the study was very small [35].

In addition, the levels of vitamin D were inversely related to BMI, AMH, peak estradiol levels, the number of larger follicles and oocytes, though this difference was not statistically significant in our study. There was a trend for worsening vitamin D status with increasing AMH, causing the increase of large follicle and oocytes. FF vitamin D levels were positively correlated with serum vitamin D levels. Recently, Antunes et al., showed similar results. Specifically, they found that lower FF vitamin D concentrations indicated a better response to ovarian stimulation shown by a greater production of larger follicles and higher serum estradiol concentrations [36]. Furthermore, FF 25 (OH) D levels were negatively correlated with AMH and AMH receptor (AMHR)-II mRNA levels in cumulus granulosa cells (GCs) [37]. Compared with women with sufficient FF 25 (OH) D levels, those with insufficient/deficient levels had a 2-fold increase in AMHR-II mRNA levels in cumulus GCs. Treatment with vitamin D3 caused a decrease in AMHR-II [37]. These data suggest that vitamin D alters AMH signaling in human cumulus GCs and plays a role in follicle development.

Our study suggests that there were no association between serum 25 (OH) D and clinical pregnancy or live birth. This is in agreement with the majority of studies [26–30, 33]. However, others have reported positive [17–24] as well as inverse associations [25, 35] between 25 (OH) D and clinical pregnancy. Therefore, whether vitamin D has a role in the outcomes of ART requires further random cohort studies to clarify. Perhaps a prospective randomized control trial would be able to remove biases and give stronger answers.

Even if our study is retrospective, there are still several strengths. The primary strength is the large number of participants. There were only 59–202 patients in previous reports [19, 25, 26, 33–36], while we observed 848 patients. Furthermore, the population in our study was homogeneous. They were all of Chinese origin. In previous papers, the population was various including Iranian [26, 27], Greek [25] and highly diverse American patients [19]. In this study, race is relevant, since it affects the relationship between vitamin D status and pregnancy rates after IVF [17]. Another strength of this study is that we excluded couples with moderate and severe male factor. We evaluated only patients undergoing IVF to minimize the role of spermatozoa affecting embryo development. In contrast, in some studies, the indications for IVF/ ICSI were not mentioned in papers [19, 25, 26]. While in other studies, 58–65% of couples had male factor [27].

Some limitations of this study must be considered though. Firstly, this study is a retrospective observational study, we cannot exclude all confounding factors. However, we adjusted the potential confounding factors for a wide range to provide reassurance of the validity of our findings. Secondly, we did not measure the vitamin D levels of couples, since serum 25 (OH) D correlates within the partners of infertile couples [38]. Moreover, we excluded couples with moderate and severe male factor and evaluated only patients undergoing IVF to minimize the role of spermatozoa affecting embryo development. Thirdly, we did not measure the vitamin D levels at the same points of IVF cycle. However, we evaluated vitamin D levels before entering the IVF cycle. To prevent confounders, women whose plasma total 25 (OH) D measurement exceeded 4 weeks prior to entering their IVF cycle were excluded from the study.

## Conclusions

In summary, we found that vitamin D was positively related to fertilization rate. However, vitamin D was unrelated to clinical pregnancy or live birth in this retrospective cohort study. In this study, patients had serum 25 (OH) D concentrations between 4.92 and 39.51 ng/ml. FF vitamin D levels were positively correlated with serum vitamin D levels. The levels of FF vitamin D were significantly higher than the levels of serum vitamin D. The relationship of vitamin D and outcomes of ART should be studied in future large randomized controlled trials.

## Additional file

Additional file 1: **Table S1.** Patients characteristics in four seasons. (DOCX 16 kb)

## Data Availability

The primary data for this study is available from patient medical records.

## Abbreviations

25 (OH) D: 25-hydroxyvitamin D
AMH: Anti-Mullerian hormone
ART: Assisted reproductive technologies
BMI: Body mass index
COH: Controlled ovarian hyperstimulation
ICSI: Intracytoplasmic sperm injection
IVF: In vitro fertilization
