# Supplementary material for: Effect of vitamin D status on normal fertilization rate following in vitro fertilization
Source: Reprod Biol Endocrinol. 2019 Jul 18;17:59. doi: 10.1186/s12958-019-0500-0 (PMC6639905; doi:10.1186/s12958-019-0500-0)
Supplement: Supplementary file 1 — Table S1. Patients characteristics in four seasons. (DOCX 16 kb) [file 12958_2019_500_MOESM1_ESM.docx]

**Additional file 1: Table S1** Patients characteristics in four seasons.

| Parameter | Spring | Summer | Autumn | Winter | P value |
| --- | --- | --- | --- | --- | --- |
| Number of cycles | 143 | 245 | 215 | 245 |  |
| 25OHD(ng/ml) | 13.41±5.62 | 14.02±5.10 | 17.55±5.90 | 15.53±5.56 | ＜0.001 |
| Age | 31.47±3.47 | 31.82±3.39 | 31.40±3.70 | 31.88±3.79 | 0.411 |
| Duration of infertility(y) | 3.43±1.99 | 3.76±2.45 | 3.77±2.57 | 3.62±2.47 | 0.536 |
| BMI(kg/m^2^) | 23.71±3.66 | 23.68±3.48 | 23.08±2.84 | 23.43±3.37 | 0.201 |
| AMH(ng/ml) | 6.85±5.88 | 6.35±4.44 | 7.15±4.87 | 6.63±5.99 | 0.423 |
| bFSH(mIU/ml) | 6.92±1.87 | 6.85±2.03 | 6.71±1.59 | 6.80±1.92 | 0.744 |

Note: *: Values are significantly different between groups (P<0.05).
